# Cephalic and saphenous venous blood collected by continuous heating of the paws compared with arterial blood for measurement of blood gas values in well‐perfused dogs

**DOI:** 10.1111/jvim.16745

**Published:** 2023-05-24

**Authors:** Yukihito Shiroshita, Ryou Tanaka, Miki Shimizu, Yoshihisa Yamane

**Affiliations:** ^1^ Department of Veterinary Surgery, Faculty of Agriculture Tokyo University of Agriculture and Technology Tokyo Japan; ^2^ VeRMS (Veterinary Respiratory Medicine and Surgery) Study Group Zama Kanagawa Japan

**Keywords:** acid‐base disturbance, arterial blood gas, arterialization, cephalic venous blood, saphenous venous blood

## Abstract

**Background:**

“Arterialization” of the dorsal hand vein is well‐established in human medicine, but not in veterinary medicine.

**Objectives:**

To compare cephalic and saphenous venous blood collected by continuously heating the paws to 37°C (“arterialization”), with arterial blood (AB) for measurement of blood gas variables in well‐perfused dogs.

**Animals:**

Eight healthy dogs.

**Methods:**

Experimental study. Fore and hind paws were continuously heated to 37°C to “arterialize” cephalic and saphenous venous blood. AB and “arterialized” cephalic and saphenous venous blood (ACV and ASV, respectively) were simultaneously collected from lightly anesthetized dogs with induced metabolic and respiratory acid‐base disorders. The pH, partial pressures of carbon dioxide (PCO_2_) and oxygen (PO_2_), bicarbonate concentration [HCO_3_^−^], and base excess (BE) were measured once in each state. Systolic blood pressure was maintained above 100 mm Hg. The AB, ACV, and ASV values were compared.

**Results:**

The pH, [HCO_3_^−^], and BE values had no significant difference and good agreement, the PCO_2_ values had a strong correlation (correlation coefficient of .91‐1.00), and the PO_2_ values had a significant difference (*P* < .01) and poor agreement between AB and ACV, and between AB and ASV. The PCO_2_ values of ASV overestimated those of AB by ~3.0 mm Hg, which was considered within clinically allowable limits, while those of ACV were not within clinically allowable limits.

**Conclusions and Clinical Importance:**

Under experimental conditions, the ASV samples were more identical to the AB samples than the ACV samples for pH, PCO_2_, [HCO_3_^−^], and BE values in well‐perfused dogs. The saphenous vein is suitable for “arterialization.”

**Abbreviations**
AB: arterial blood
ACV: arterialized cephalic venous blood
ASV: arterialized saphenous venous blood
BE: base excess
FIO_2_
: fraction of inspiratory oxygen
PCO_2_
: partial pressure of carbon dioxide
PO_2_
: partial pressure of oxygen
[HCO_3_^−^]: bicarbonate concentration

## 1 INTRODUCTION

Venous blood samples have been used to estimate acid‐base balance in human^1^, ^2^, ^3^, ^4^, ^5^, ^6^, ^7^, ^8^, ^9^ and veterinary medicine,^10^, ^11^, ^12^, ^13^ instead of arterial blood (AB) samples. However, because of its inaccuracy, venous blood gas analysis might be inadequate for clinical decision‐making.^14^, ^15^

In human medicine, “arterialization” of the dorsal hand vein is a well‐established blood sampling technique. In well‐perfused humans, heating hands to 42°C to 43°C for 10 to 15 minutes^16^, ^17^, ^18^ causes venous blood to become more similar to AB.^1^, ^19^ This technique allows sample collection without venous stasis and with the patient lying in bed.^1^, ^20^ Therefore, “arterialization” is an easy, safe, and noninvasive technique for AB gas sampling. In humans, “arterialized” venous blood can substitute AB for the measurement of pH, partial pressure of carbon dioxide (PCO_2_), and lactate.^1^, ^16^, ^20^, ^21^

In modern human medicine, “arterialization” has only been applied to volunteer subjects participating in medical research.^17^, ^22^, ^23^ Because this time‐consuming procedure before sampling prevents collecting samples when they are urgently needed, it is rarely used in clinical practice. Furthermore, heating the hand to temperatures above 42°C might be uncomfortable for some patients.^1^, ^21^ However, adequate “arterialization” can be achieved as long as the cutaneous temperature remains above 35°C to 37°C.^1^, ^17^ To our knowledge, only 1 experimental study has documented the use of “arterialized” venous blood in dogs,^24^ in which the cephalic vein was “arterialized” by continuously heating the dogs' forepaws to 40°C using an incandescent lamp. It might be possible to collect blood as necessary without causing discomfort by continuously heating the forepaw to a relatively low cutaneous temperature of 35°C to 37°C.

“Arterialized” peripheral venous blood gas analysis might be useful for repeated blood gas sampling in animals with severe uremic acidosis and diabetic ketoacidosis,^25^ as well as in animals under postoperative observation. In veterinary clinical settings, the cephalic vein is often used for intravenous infusions and is not always an appropriate site for “arterialized” venous blood sampling. The saphenous vein in the hind leg, which is used for venous blood sampling as an alternative to the cephalic vein, is a preferred site for “arterialized” venous blood sampling. However, information on “arterialization,” including suitable sites for venous blood sampling, is lacking in veterinary medicine.

The present study aimed to compare cephalic and saphenous venous blood collected by continuously heating the paws to 37°C (“arterialization”) with AB for the blood gas and acid‐base values in well‐perfused dogs with metabolic and respiratory acid‐base disorders.

## 2 MATERIALS AND METHODS

### 2.1 Animals

This study was approved by the institutional laboratory animal care and use committee of the study institute. In this study, eight clinically healthy adult crossbreed dogs (4 males and 4 females; KITAYAMA LABES CO., LTD., Nagano, Japan) were used. Their median age was 2.0 (range, 2.0‐6.0) years; their median body weight was 8.5 (range, 7.6‐13.1) kg; their median packed cell volume was 39.4% (range, 34.5%‐52.7%); and their median hemoglobin concentration was 13.9 (range, 12.2‐19.1) g/dL. Physical examination, chest X‐rays, a complete blood count, and a blood biochemistry profile revealed that all of the dogs used in the study showed no indication of cardiovascular and respiratory ailments.

### 2.2 Study design

**FIGURE 1:**
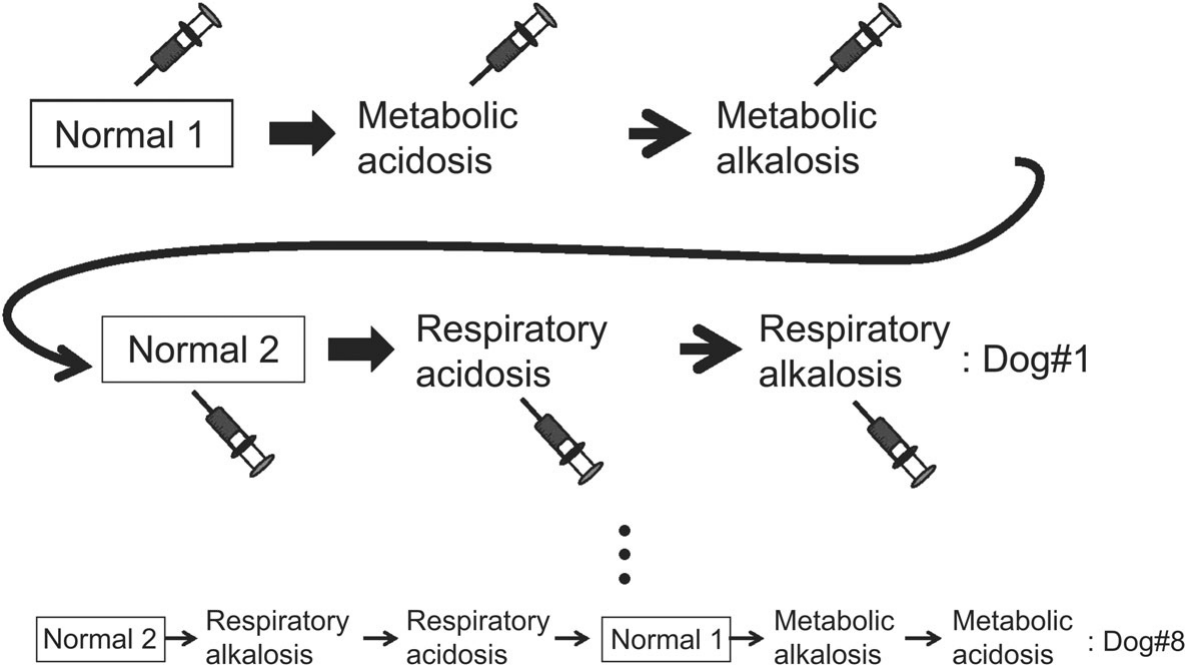
Repeated measures and a randomized block design in the present study. All types of acid‐base disorders were experimentally induced. Ten minutes before producing metabolic or respiratory acid‐base disorders, the acid‐base state was controlled normally. Blood samples were collected once in each state. It took 1 to 3 hours to wash out between these acid‐base states. These six states could be randomly arranged in eight blocks. One dog was allocated to each block. Therefore, eight experiments were completely performed.

The dogs were lightly anesthetized and maintained in a normal metabolic and respiratory state while breathing air until the first experiment, in which metabolic acidosis and alkalosis were induced. Following appropriate intervals to allow the washout of induced acid‐base disorders, the dogs were allowed to return to their normal acid‐base state before the second experiment, in which respiratory acidosis and alkalosis were experimentally induced. Similarly to the first experiment, appropriate intervals were maintained in the second experiment to allow washout of the acid‐base disturbed states, and the dogs were allowed to return to their normal state. The order of the induced acid‐base states was randomized (randomized block design).^26^ The present study consisted of eight blocks that were patterns of orders of induced acid‐base states, with one dog allocated to each block; thus, a minimum of eight dogs were required in the present study (Figure 1). Before the experiments, two preliminary experiments were performed in two normal, healthy adult dogs to standardize the study protocol and washout intervals based on a previous study's protocol.^24^ Steady heat was applied to the fore and hind paws of the dogs using two incandescent lamps set 10 to 15 cm lateral to the paws to maintain a cutaneous temperature of 37°C to intend to “arterialize” cephalic and saphenous venous blood. AB, “arterialized” cephalic venous blood (ACV), and “arterialized” saphenous venous blood (ASV) samples were collected simultaneously once in each acid‐base state and analyzed for pH, PCO_2_, partial pressure of oxygen (PO_2_), bicarbonate concentration ([HCO_3_^−^]), and base excess (BE). The values of the ACV and ASV samples were compared with those of the AB samples. The overall ambient temperature of the experimental setup was maintained between 22°C and 24°C. During blood sampling, three co‐experimenters, all of whom were professional veterinary practitioners, were randomly allocated to each sampling site. They were not informed about the interim report on experimental results during the experiments.

### 2.3 Anesthesia and maintenance

All dogs were pretreated with acetylpromazine (0.1 mg/kg IM), butorphanol (0.2 mg/kg IM), and atropine (0.05 mg/kg IM), followed by anesthesia with thiamylal (10 mg/kg IV). The dogs were intubated and allowed to breathe ambient air while lying in lateral recumbency on an electric heating blanket. Based on previous studies,^27^, ^28^, ^29^, ^30^ a continuous infusion of ketamine (10‐60 μg/kg/min), pentobarbital (5 mg/kg IV, appropriately q1‐3h), and butorphanol (.05 mg/kg IM, q1h) were administered to ensure the dogs were on a light plane of anesthesia throughout the experiments without affecting cardiorespiratory function or losing eyelid reflex. The infusion was maintained via a cephalic vein that was not used to collect blood samples during the experiments. Two thermometer probes were surgically inserted into each dog's fore and hind paws to record cutaneous temperature. The oxygen saturation (SpO_2_) was measured with a pulse oximeter (CAPNOX, Colin, Aichi, Japan), the breathing rate and end‐tidal PCO_2_ were measured with a side‐stream capnometer (CAPNOX, Colin, Aichi, Japan), and the AB pressure was measured with an oscillometric blood pressure device (Dynamap 8300, Critikon, Tampa, FL). The heart rate and rhythm, as well as the rectal and esophageal temperatures, were continuously monitored. When the dogs returned to a steady state, the baseline values of all variables and the tidal volume were recorded using a respirometer (RM121, Citizen, Yamanashi, Japan). SpO_2_ was monitored and maintained at or above 90%. To avoid severe hypoxemia, the dogs were given 100% oxygen if their SpO_2_ fell under 90%. Systolic arterial pressure and heart rate were maintained above 100 mm Hg and between 70 and 150 beats/min, respectively, by adjusting the rate of ketamine infusion, as appropriate. Rectal and esophageal temperatures were maintained around 38°C by adjusting the temperature of the electric heating blanket.

### 2.4 Blood sample collection, handling, and measurement

AB samples were collected from the femoral artery in heparinized syringes through a 22‐gauge catheter with an extension tube and a 3‐way stopcock. ACV and ASV samples were collected from the distal part of the limb via venipuncture with minimal venous stasis to avoid blood flow obstruction. All three blood samples (2 mL each) were obtained anaerobically. Before blood sampling, vital signs, SpO_2_, and end‐tidal PCO_2_ were maintained stable and steady for at least 5 minutes. The syringe tubes were immediately and securely capped, placed on ice, and analyzed within 10 minutes in a random sequence on a blood gas analyzer^31^ (AVL Scientific, Roswell, GA) at 37°C. To ensure accuracy, the analyzer was calibrated before each experiment using control acidic, normal, and alkaline solutions (CONFITEST III, AVL Scientific), and the measurements were kept within permissible target limits of ±0.04 units for pH; ±3 mm Hg for PCO_2_; and ±3, ±8, and ±15 mm Hg for PO_2_ values of 68, 107, and 147 mm Hg, respectively.

### 2.5 Experiment 1: Metabolic acid‐base disorders

All arterial variables were confirmed to be within the normal range before any experiments. Based on these initial baseline values, mechanical ventilation (Compos β‐EV, Silver Medical, Tokyo, Japan) with compressed air (Kawasaki Sogo Gas Center, Kawasaki, Japan) was initiated at a frequency of 8 to 16 breaths/min and a tidal volume of 10 to 25 mL/kg. This state, that is, Normal 1, was maintained for 10 minutes before simultaneous blood sampling. After sampling, the dogs were allowed to breathe ambient air without mechanical ventilation.

Metabolic acidosis was induced by infusing 0.6‐M ammonium chloride (NH_4_Cl; Conclyte‐A, Hishiyama Pharmaceutical, Osaka, Japan) in a 5% dextrose solution at a rate of 3.5 to 6.5 mEq/kg/h for 60 to 100 minutes before simultaneous blood sampling. To induce metabolic alkalosis, 1 M sodium bicarbonate (NaHCO_3_; MEYLON84, Otsuka Pharmaceutical Factory Inc., Tokushima, Japan) was infused at a rate of 5 to 10 mEq/kg/h for 60 to 100 minutes before simultaneous blood sampling. During Experiment 1, the acid‐base state was monitored, and AB gases were measured at 15‐minute intervals. To allow for washout, a 2‐hour interval was allowed between the metabolic acidosis and metabolic alkalosis states. All dogs were allowed to recover from metabolic acidosis and metabolic alkalosis with appropriate treatments.

### 2.6 Experiment 2: Respiratory acid‐base disorders

Normoxic ventilation was established, and blood samples from the three sites were collected simultaneously in this state, that is, Normal 2, as described in Experiment 1. Next, respiratory acidosis was induced by mechanical ventilation with 100% oxygen at a frequency of 2 to 4 breaths/min and a tidal volume of 15 to 25 mL/kg. To impede hypoxemia, 100% oxygen was used. To induce apnea and adjust the respiratory rate, succinylcholine (2 mg/head IV; Succin, Yamanouchi Pharmaceutical, Tokyo, Japan) and pentobarbital (2.5 mg/kg IV) were administered at 15‐minute intervals. Blood samples were collected after a 60‐minute treatment period. To induce respiratory alkalosis, mechanical ventilation with compressed air at a frequency of 10 to 16 breaths/min and a tidal volume of 25 to 50 mL/kg was used. Mechanical ventilation was maintained for 60 minutes before blood sampling. To maintain good perfusion, half saline (saline:5% dextrose, 1:1) was infused at a rate of 5 to 10 mL/kg/h throughout Experiment 2.

When Experiment 1 was followed by Experiment 2, the dogs were given a 3‐hour washout interval. When Experiment 2 was followed by Experiment 1, the dogs were given a 1‐hour washout interval. Following all experimental procedures, the dogs were allowed to extubate and awaken from anesthesia.

### 2.7 Statistical analysis

To determine the validity of the washout, mean differences in the values of all arterial variables between the Normal 1 and 2 states were calculated, and the arterial values between these two states were compared using a two‐tailed paired Student's *t‐*test.

The mean values of all variables in arterial and “arterialized” venous blood samples during all the induced acid‐base states were compared using a multivariate approach to repeated‐measures analysis of variance (MANOVA).^32^ The strength of the linear relationship between arterial and “arterialized” venous blood sample variables during induced metabolic and respiratory acid‐base states was examined using linear regression analysis, and correlation coefficients (*R*) and coefficients of determination (*R* ^2^) were calculated. The degree of agreement between arterial and “arterialized” venous blood sample variables was assessed by plotting the differences between the two measurements against their average (ie, Bland‐Altman plots^33^). The PO_2_ data were divided into two groups of states during oxygen inhalation at fraction of inspiratory oxygen (FIO_2_) values of 1.0 and 0.21, which have a significant impact on PO_2_. All data were analyzed using the Statistical Package for the Social Sciences (IBM SPSS Statistics, Version 28.0, Armonk, NY). A *P‐*value of <.05 was considered statistically significant.

In the present study, clinical agreement was determined by defining the clinically allowable limits for each variable on Bland‐Altman plots using the following criteria required to pass proficiency testing for quality control^2^, ^34^, ^35^, ^36^: 80% of the data points should be within ±0.04 for pH, ±5.00 mm Hg for PCO_2_, and ±15.51 mm Hg for PO_2_ on a standard analyzer.^35^, ^36^ The clinically allowable limits for [HCO_3_^−^] were set at ±1.50 mmol/L to account for the analyzer's accuracy.^31^

## 3 RESULTS

### 3.1 Heated‐paw technique

At the beginning of the experiments, the median cutaneous temperature of the fore and hind paws was 35.2°C (range, 32.1°C‐37.0°C) before applying the heat lamp, and the paws were heated to a cutaneous temperature of 37°C using incandescent lamps to intend to “arterialize” the cephalic and saphenous venous blood. The cutaneous temperature measured by thermometers surgically inserted into the paws was maintained at a median of 37.2°C (range, 36.5°C‐39.1°C) with the lamps for a median of 464 (range, 400‐541) minutes. The decrease in cutaneous temperature was confirmed by palpation without the use of a thermometer at the time of extubation, which was 60 to 80 minutes after removal of the lamps. No late complications were observed at the 1‐week follow‐up after recovery from anesthesia, and no temperature‐related burns were observed in any of the dogs.

### 3.2 Washout periods

The mean differences in arterial pH, PCO_2_, PO_2_, [HCO_3_^−^], and BE during the Normal 1 and 2 states were −0.018 ± 0.036 (*P* = .20), 0.0 ± 3.8 mm Hg (*P* > .99), 3.5 ± 12.7 mm Hg (*P* = .46), −0.9 ± 1.2 mmol/L (*P* = .071), and −1.1 ± 1.3 mmol/L (*P* = .046), respectively. No significant differences were detected between the two states, except for BE. The mean differences in BE were clinically insignificant. Therefore, the washout intervals were considered valid.

### 3.3 Vital signs and AB pressure

During this experiment, the heart and respiratory rates, rectal and esophageal temperatures, systolic blood pressure, and AB pressure were 127 (range, 65‐225) beats/min, 15 (range, 1‐81) breaths/min, 38.4°C (range, 35.3°C‐39.6°C), 37.8°C (range, 35.3°C‐39.4°C), 116 (range, 79‐211) mm Hg, and 87 (range, 50‐138) mm Hg, respectively.

### 3.4 pH, PCO_2_ , [HCO_3_ ^−^], and BE

The blood gas values measured in all eight dogs in the present study were analyzed without exclusion. Among the AB, ACV, and ASV samples, the pH, PCO_2_, [HCO_3_^−^], and BE values showed no statistically significant differences in all acid‐base states, except for PCO_2_ values under metabolic acidosis and pH and PCO_2_ values under the Normal 2 state (Table 1). The PCO_2_ values of the ACV and ASV under metabolic acidosis were significantly higher (*P* < .01 and *P* < .05, respectively) than those of the AB (Table 1).

**TABLE 1:** Blood gases and acid‐base measurements (mean ± SD) in dogs with induced acid‐base disturbances (n = 8)

Variables	Acid‐base status		AB	ACV	ASV
pH (unit)	Normal 1^a^		7.374 ± 0.044	7.352 ± 0.036	7.350 ± 0.046
	Met. acidosis		7.213 ± 0.070	7.186 ± 0.058	7.203 ± 0.064
	Met. alkalosis		7.481 ± 0.043	7.460 ± 0.043	7.460 ± 0.047
	Normal 2^a^		7.392 ± 0.019	7.366 ± 0.017*	7.368 ± 0.024
	Res. acidosis		7.203 ± 0.053	7.180 ± 0.054	7.187 ± 0.053
	Res. alkalosis		7.488 ± 0.029	7.468 ± 0.034	7.468 ± 0.030
PCO_2_ (mm Hg)	Normal 1^a^		37.7 ± 3.1	40.2 ± 2.8	40.4 ± 3.3
	Met. acidosis		33.2 ± 3.0	38.7 ± 2.6**	37.5 ± 2.6*
	Met. alkalosis		43.6 ± 3.8	47.6 ± 4.9	46.8 ± 4.3
	Normal 2^a^		37.7 ± 2.4	41.3 ± 3.0*	40.9 ± 2.2
	Res. acidosis		62.3 ± 9.4	68.0 ± 10.0	65.6 ± 9.8
	Res. alkalosis		25.4 ± 3.5	28.3 ± 3.1	28.6 ± 3.9
PO_2_ (mm Hg)	Normal 1^a^	FIO_2_ 0.21, n = 8	80.6 ± 7.6	55.0 ± 4.5**	56.7 ± 7.1**
	Met. acidosis	FIO_2_ 0.21, n = 7	95.0 ± 16.3	53.5 ± 6.8**	59.2 ± 6.2**
		FIO_2_ 1.0, n = 1	528.4	79.0	249.0
	Met. alkalosis	FIO_2_ 0.21, n = 7	83.7 ± 5.3	52.5 ± 5.3**	52.9 ± 6.1**
		FIO_2_ 1.0, n = 1	571.5	55.6	57.4
	Normal 2^a^	FIO_2_ 0.21, n = 8	77.1 ± 10.4	52.4 ± 10.9**	54.4 ± 10.7**
	Res. acidosis	FIO_2_ 0.21, n = 0	ND	ND	ND
		FIO_2_ 1.0, n = 8	554.0 ± 20.6	154.8 ± 95.8**	235.5 ± 140.0**
	Res. alkalosis	FIO_2_ 0.21, n = 8	87.3 ± 11.6	48.6 ± 8.8**	51.6 ± 6.7**
		FIO_2_ 1.0, n = 0	ND	ND	ND
[HCO_3_^−^] (mmol/L)	Normal 1^a^		21.5 ± 1.5	21.8 ± 1.5	21.8 ± 1.7
	Met. acidosis		13.2 ± 2.5	14.5 ± 2.5	14.5 ± 2.4
	Met. alkalosis		31.9 ± 4.1	33.3 ± 5.0	32.7 ± 4.6
	Normal 2^a^		22.4 ± 1.3	23.1 ± 1.3	23.0 ± 1.3
	Res. acidosis		24.0 ± 3.8	24.8 ± 4.1	24.3 ± 3.7
	Res. alkalosis		18.8 ± 2.1	19.9 ± 1.3	20.2 ± 1.8
BE (mmol/L)	Normal 1^a^		−3.1 ± 2.0	−3.4 ± 1.8	−3.4 ± 2.2
	Met. acidosis		−13.3 ± 3.5	−12.8 ± 3.1	−12.6 ± 3.3
	Met. alkalosis		7.5 ± 3.9	7.9 ± 4.5	7.6 ± 4.3
	Normal 2^a^		−2.0 ± 1.2	−2.0 ± 1.0	−2.0 ± 1.4
	Res. acidosis		−5.1 ± 3.4	−5.0 ± 3.7	−5.3 ± 3.4
	Res. alkalosis		−2.6 ± 1.8	−2.1 ± 1.6	−2.0 ± 1.3

Abbreviations: AB, arterial blood; ACV, arterialized cephalic venous blood; ASV, arterialized saphenous venous blood; Met., metabolic; Res., respiratory.

^a^ Refer to Figure 1.

* Significantly different (*p* < .05) from AB.

** Significantly different (*p* < .01) from AB.

**FIGURE 2:**
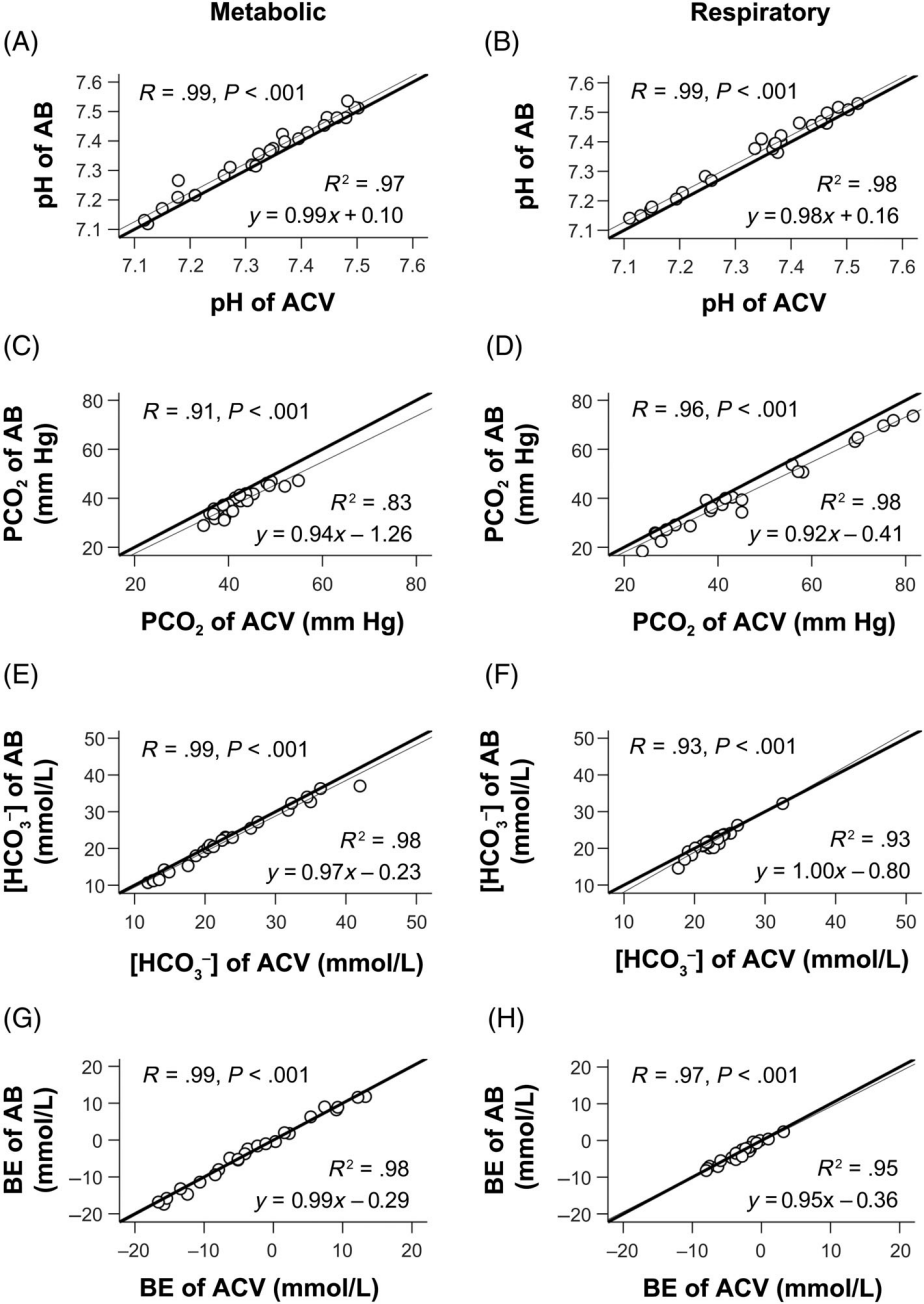
The linear regression analyses demonstrate that the pH (A,B), PCO_2_ (C,D), [HCO_3_^−^] (E,F), and BE (G,H) values of the AB and ACV samples were strongly correlated. The two measurements of AB and ACV samples were nearly identical under both metabolic (A,C,E,G) and respiratory (B,D,F,H) acid‐base disorders in all eight dogs. The thick lines indicate that the two measurements of the AB and ACV samples are identical. The thin lines are regression lines. AB, arterial blood; ACV, arterialized cephalic venous blood; BE, base excess; PCO_2_, partial pressure of carbon dioxide; *R*, correlation coefficient; *R* ^2^, coefficient of determination; [HCO_3_^−^], bicarbonate concentration.

**FIGURE 3:**
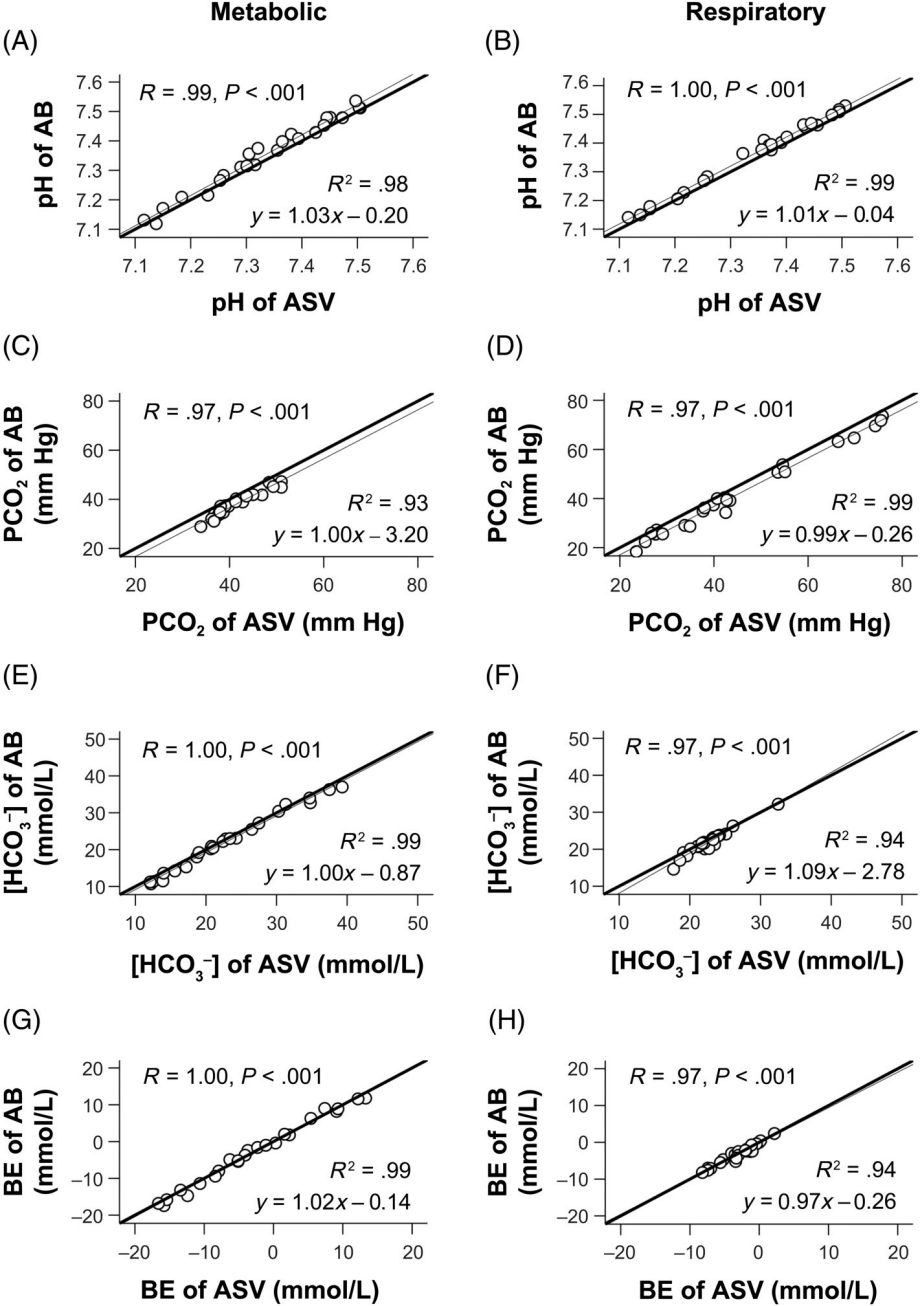
The linear regression analyses demonstrate that the pH (A,B), PCO_2_ (C,D), [HCO_3_^−^] (E,F), and BE (G,H) values of the AB and ASV samples were very strongly correlated and are almost identical under both metabolic (A,C,E,G) and respiratory (B,D,F,H) acid‐base disorders in all eight dogs. The thick lines indicate that the two measurements of the AB and ASV samples are identical. The thin lines are regression lines. AB, arterial blood; ASV, arterialized saphenous venous blood; BE, base excess; PCO_2_, partial pressure of carbon dioxide; *R*, correlation coefficient; *R* ^2^, coefficient of determination; [HCO_3_^−^], bicarbonate concentration.

Under both metabolic and respiratory acid‐base states, the correlation and linear regression analyses revealed that the pH, PCO_2_, [HCO_3_^−^], and BE values of the ACV and ASV samples had strong, positive correlations with those of the AB samples (*R* = .91‐1.00; *R* ^2^ = .83‐.99), with a slope close to 1.0 and a *y*‐intercept close to zero (Figures 2 and 3). During metabolic acid‐base states, PCO_2_ values of the ACV samples had a relatively lower degree of correlation with those of the AB samples (*R* = .91; *R* ^2^ = .83, slope: 0.93; Figure 2C), whereas PCO_2_ values of the ASV samples had a strong, positive correlation with those of the AB samples (*R* = .97; *R* ^2^ = .93; slope: 1.0; Figure 3C).

**FIGURE 4:**
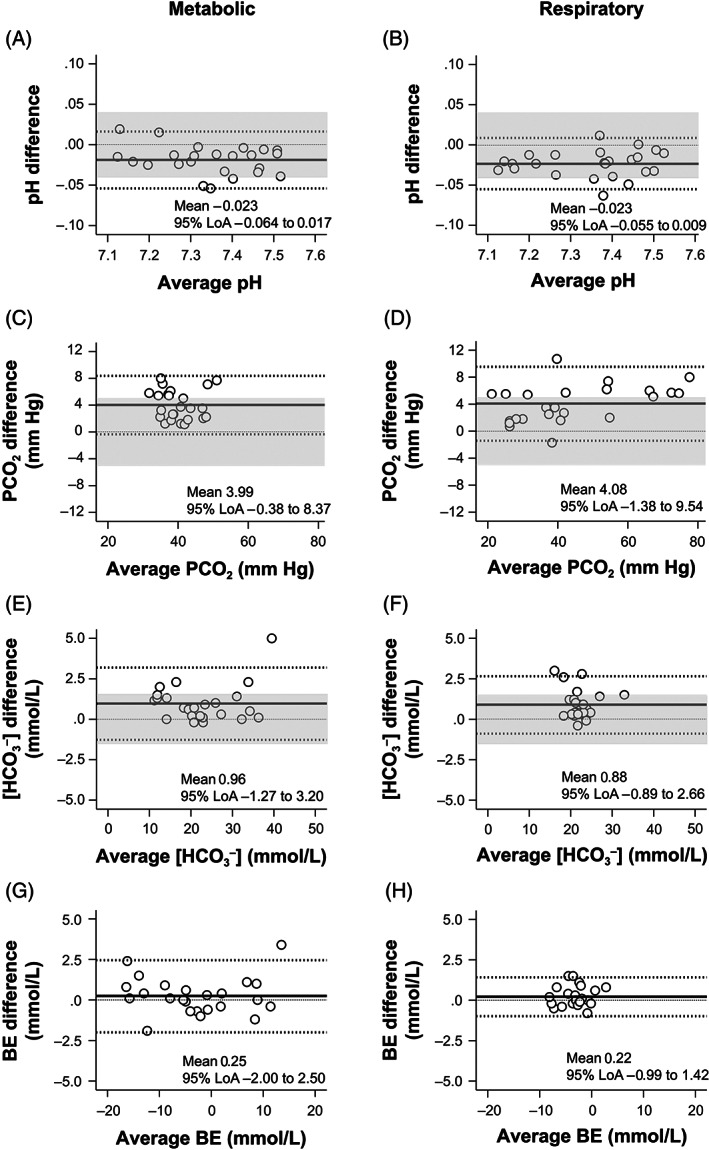
The Bland‐Altman plots for pH (A,B), PCO_2_ (C,D), [HCO_3_^−^] (E,F), and BE (G,H) values show differences between the two measurements of the AB and ACV samples against their average under metabolic (A,C,E,G) and respiratory (B,D,F,H) acid‐base disorders. The central solid line represents the mean bias of measurements; the adjacent broken lines show the 95% LoA (±1.96 SD); and the thin dashed line is the best fit. The clinically allowable limits defined in the present study are shaded gray. The pH, [HCO_3_^−^], and BE values of the ACV samples showed good clinical agreement with those of the AB samples. The PCO_2_ values of the ACV overestimated those of the AB (mean bias of 3.99 and 4.08 mm Hg, respectively) under metabolic and respiratory acid‐base disorders (C,D). AB, arterial blood; ACV, arterialized cephalic venous blood; BE, base excess; PCO_2_, partial pressure of carbon dioxide; [HCO_3_^−^], bicarbonate concentration; 95% LoA, 95% limits of agreement.

The bias and 95% limits of agreement on the Bland‐Altman plots for pH, [HCO_3_^−^], and BE of the ACV samples showed good clinical agreement with those of the AB samples (Figure 4A,B,E,F,G,H). During metabolic and respiratory acid‐base states, 80% of the data points on the Bland‐Altman plots for pH and [HCO_3_^−^] of the ACV samples were within clinically allowable limits (83.3%‐87.5%). PCO_2_ values of ACV samples overestimated those of AB samples in both metabolic and respiratory acid‐base states (mean bias, 3.99 and 4.08 mm Hg, respectively; Figure 4C,D), and the data points for PCO_2_ of ACV samples during metabolic and respiratory acid‐base states were not within clinically allowable limits (62.5% and 50.0%, respectively; Figure 4C,D).

**FIGURE 5:**
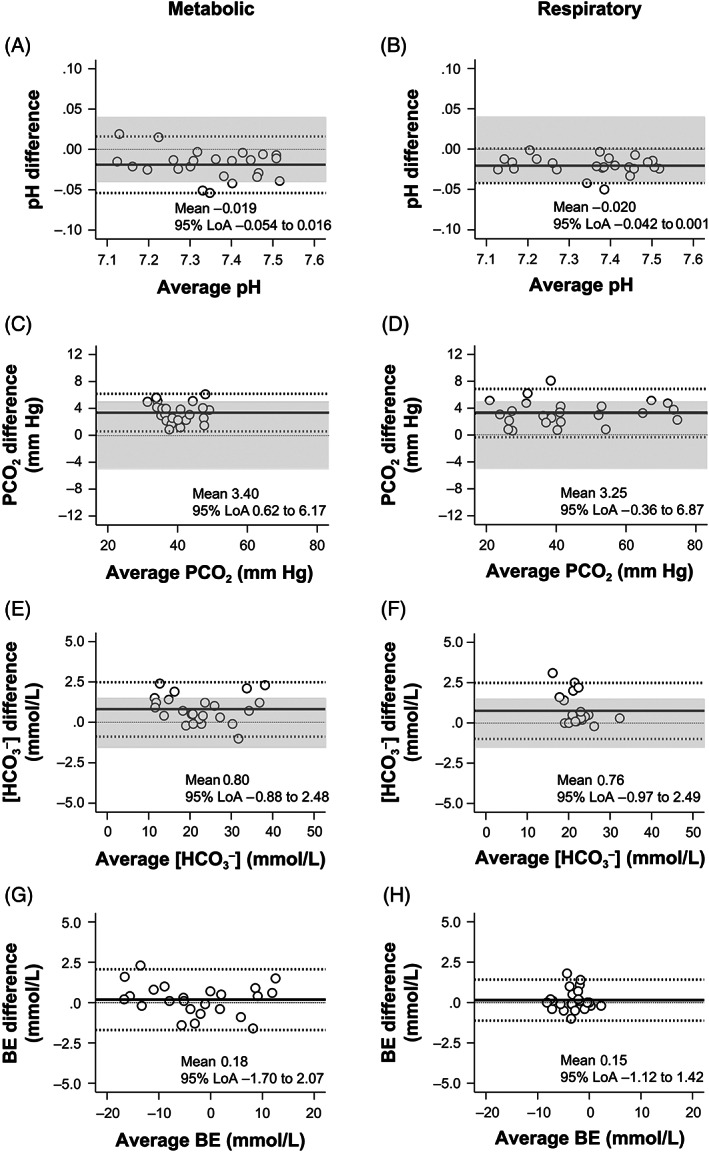
The Bland‐Altman plots for pH (A,B), PCO_2_ (C,D), [HCO_3_^−^] (E,F), and BE (G,H) values show differences between the two measurements of the AB and ASV samples against their average under metabolic (A,C,E,G) and respiratory (B,D,F,H) acid‐base disorders. The central solid line represents the mean bias of measurements; the adjacent broken lines show the 95% LoA (±1.96 SD); and the thin dashed line is the best fit. The clinically allowable limits defined in the present study are shaded gray. The pH, [HCO_3_^−^], and BE values of the ASV samples showed good clinical agreement with those of the AB samples. The PCO_2_ values of the ASV overestimated those of the AB (mean bias of 3.40 and 3.25 mm Hg, respectively) under metabolic and respiratory acid‐base disorders (C, D). AB, arterial blood; ASV, arterialized saphenous venous blood; BE, base excess; PCO_2_, partial pressure of carbon dioxide; [HCO_3_^−^], bicarbonate concentration; 95% LoA, 95% limits of agreement.

The Bland‐Altman plots for pH, [HCO_3_^−^], and BE of the ASV samples showed good clinical agreement with those of the AB samples, and the data points for pH and [HCO_3_^−^] of the ASV samples were within clinically allowable limits (83.3%‐91.7%; Figure 5A,B,E,F,G,H). Although the PCO_2_ values of the ASV samples slightly overestimated those of the AB samples in both metabolic and respiratory acid‐base states (mean bias, 3.40 and 3.25 mm Hg, respectively; Figure 5C,D), 83.3% of the data points were within clinically allowable limits in both metabolic and respiratory acid‐base states (Figure 5C,D).

### 3.5 PO_2_

**FIGURE 6:**
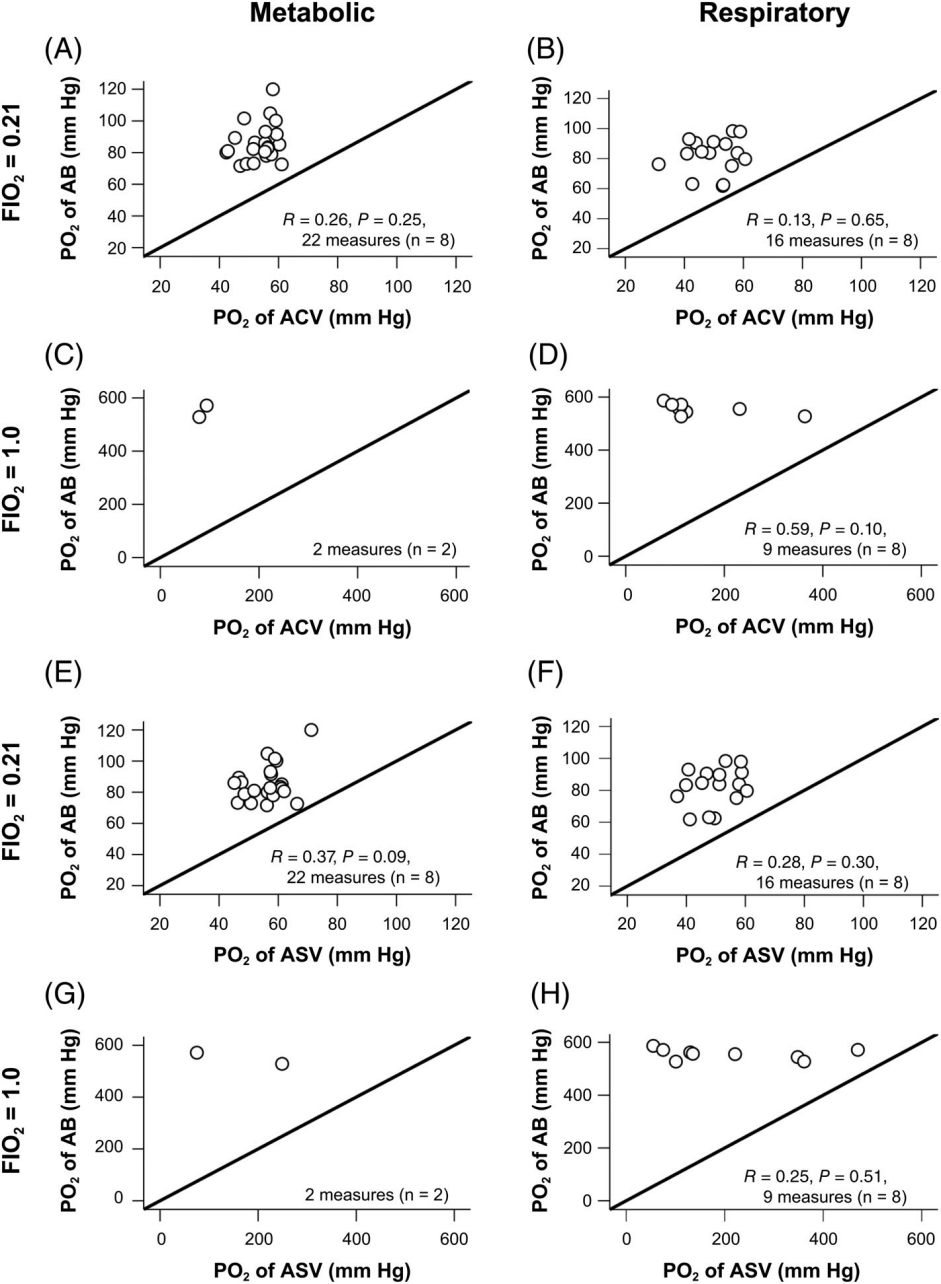
The linear regression analyses show that the PO_2_ values of the AB and ACV (A‐D), as well as the AB and ASV (E‐H) samples, had no correlation during O_2_ inhalation at FIO_2_ of 0.21 (A,B,E,F) and FlO_2_ of 1.0 (C,D,G,H). Thick lines indicate that the two measurements of the AB and ACV, as well as the AB and ASV samples, are identical. AB, arterial blood; ACV, arterialized cephalic venous blood; ASV, arterialized saphenous venous blood; FIO_2_, fraction of inspiratory oxygen; PO_2_, partial pressure of oxygen; *R*, correlation coefficient.

**FIGURE 7:**
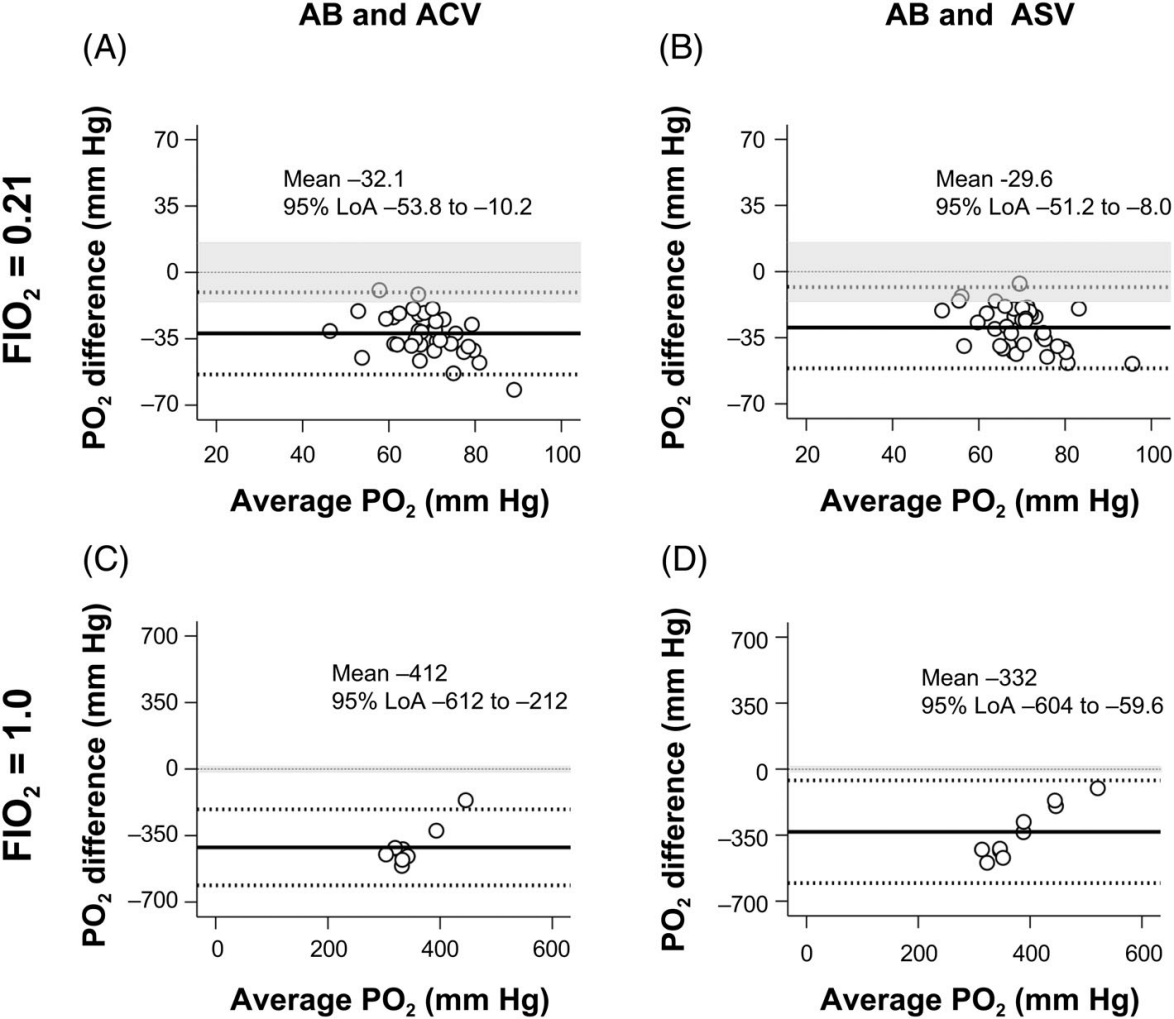
The Bland‐Altman plot analyses of the PO_2_ differences against the PO_2_ averages between the two measurements of the AB and ACV (A,C) and the AB and ASV (B,D) samples during O_2_ inhalation at FIO_2_ of 0.21 (A,B) and FIO_2_ of 1.0 (C,D). The central solid line represents the mean bias of measurements; the adjacent broken lines show the 95% LoA (±1.96 SD), and the thin dashed line is the best fit. The clinical allowable limits defined in the present study are shaded in gray. The PO_2_ values of the ACV and ASV samples showed no clinical agreement with those of the AB samples during O_2_ inhalation at FIO_2_ of 0.21 and FIO_2_ of 1.0. AB, arterial blood; ACV, arterialized cephalic venous blood; ASV, arterialized saphenous venous blood; FIO_2_, fraction of inspiratory oxygen; PO_2_, partial pressure of oxygen; 95% LoA, 95% limits of agreement.

PO_2_ values in both the ACV and ASV blood samples differed significantly from those in the AB samples (*P* < .01; Table 1). The measurements did not correlate, and the data points were not within the clinically allowable limits (4.2%‐8.3%) with AB during O_2_ inhalation at FIO_2_ of 0.21 and 1.0 under all acid‐base states (Figures 6 and 7).

## 4 DISCUSSION

The present study found good agreement of ACV and ASV, with AB in pH, [HCO_3_^−^], and BE values, while significant difference and poor agreement between AB, ACV, and ASV in PO_2_ values. In the induced metabolic acidosis state, the PCO_2_ values of ACV and ASV were significantly different from those of AB. However, a strong correlation was found in PCO_2_ values between AB and ACV and between AB and ASV in any acid‐base state, and the PCO_2_ values of ASV were in agreement with those of AB within clinically allowable limits.

Many previous studies comparing peripheral venous blood gas values to arterial and peripheral blood gas values in human^2^, ^3^, ^5^, ^6^, ^7^, ^8^, ^9^, ^37^, ^38^, ^39^, ^40^, ^41^ and veterinary medicine^10^, ^11^, ^12^, ^13^, ^42^, ^43^, ^44^, ^45^, ^46^ reported sufficient correlations despite statistically significant differences, and that venous pH, PCO_2_, and [HCO_3_^−^] measurements are in clinically acceptable agreement with arterial values if the circulatory status is not impaired. However, these authors suggested that peripheral venous blood samples are not a substitute for AB samples, even when assessing pH, PCO_2_, and [HCO_3_^−^].^11^, ^12^, ^13^ The normal arteriovenous pH difference in dogs is up to 0.04,^25^ and venous PCO_2_ values are roughly 4 to 6 mm Hg higher than arterial PCO_2_ values, even in stable states.^11^, ^43^, ^47^ In addition, the arteriovenous PCO_2_ differences vary extensively, depending on various conditions and diseases, which affect local metabolism and tissue perfusion.^14^, ^15^

Recent human clinical studies^2^, ^6^ demonstrated the limitations of using peripheral venous blood for blood gas and acid‐base assessments, especially peripheral venous PCO_2_, which is subject to local metabolism and tissue perfusion.

When evaluating acid‐base and blood gases, AB gas samples obtained via needle puncture or catheterization are preferred over venous blood samples in clinical decision‐making.^15^, ^43^ However, arterial puncture might be associated with complications, such as pain,^15^ extensive ecchymoses,^31^ hematoma,^15^, ^48^, ^49^, ^50^ peripheral nerve damage.^15^, ^48^, ^50^, ^51^ Arterial catheterization is also at risk of hemorrhage,^15^ thrombosis,^15^ infection,^15^ and, although rarely, severe vascular occlusion^15^ or fatal arterial rupture.^52^ Both arterial puncture and catheterization are difficult to perform in small or obese animals.^31^, ^52^ “Arterialization” is a fascinating theory for obtaining venous samples similar to arterial samples with safety and ease, but little is known about this procedure in veterinary medicine. The present results and technical considerations are discussed.

The AB gas and acid‐base values in the present study are similar to those reported in previous studies in healthy, awake dogs,^11^, ^12^ indicating that the experimental paradigm used in the present study provided an environment that closely resembled that of dogs in the awake state. In general, the average PCO_2_ value is around 40 mm Hg in normal humans and slightly lower in dogs (37 mm Hg).^47^ The present study results also supported these findings.

“Arterialization” is based on two mechanisms: central thermoregulation in the hypothalamus and the minimization of the influence of peripheral tissue metabolism.^20^, ^21^ Blood flow through the hand increases rapidly as the surrounding temperature rises between 35°C and 45°C. In addition, blood flow is affected by the tone of the cutaneous vessels, which is maintained by central thermoregulation, resulting in a twofold or larger difference in the hand flow depending on whether the subject is warm or cool.^21^ This is effective in areas with little muscle mass, such as the hand, potentially ensuring limited tissue metabolism.^53^ Human studies revealed that heating of the palm or foot to 37°C to 45°C before venous blood sampling provides a valid substitute for arterial pH and PCO_2_ for clinical purposes, but not arterial PO_2_.^1^, ^16^, ^22^, ^53^, ^54^

“Arterialization” using the heated‐paw technique in the present study is also considered to minimize the local tissue metabolic effect. Our findings are consistent with those of human studies.^1^, ^16^, ^22^, ^53^, ^54^ Our findings are also consistent with those of a study on dogs, which found a high correlation of pH, PCO_2_, and [HCO_3_^−^] values between the AB and ACV samples.^24^

In the present study, PCO_2_ values of both the ACV and ASV samples were significantly different from those of the AB samples under the metabolic acidosis state, despite a considerable degree of correlation with the AB samples across the range of metabolic acid‐base states. In in vitro studies, NH_4_Cl influences the vasoconstriction of isolated arteries, inducing some vasocontractile responses that might hinder the vasodilation induced by heating effects.^55^, ^56^ A previous study^24^ showed that venous PCO_2_ values under NH_4_Cl‐induced metabolic acidosis had a relatively low correlation with arterial PCO_2_ across all acid‐base states. However, PCO_2_ values of the ASV samples exhibited a strong, positive correlation with arterial PCO_2_ values under NH_4_Cl‐induced metabolic acidosis in the present study.

The present study revealed a strong, positive correlation of PCO_2_ between the AB and ASV samples compared with that between the AB and ACV samples. Since the blood sampling site of the ASV is anatomically more distal to the limb than that of the ACV, the ASV is less affected by tissue metabolism than the ACV. In humans, the veins on the back of the hand are commonly used to collect “arterialized” blood. These veins drain tissues with low metabolism (primarily skin and bone) and are easily dilated by warmth to increase blood flow through the hand.^20^ In dogs, the saphenous veins also meet this criterion.

Hypovolemia or anemia increases arteriovenous differences in acid‐base states and affects blood gas values.^13^, ^57^ A previous study^44^ found that maintaining systolic blood pressure above 80 mm Hg provided peripheral perfusion sufficient to arterialize capillary blood. In the present study, the systolic AB pressure level was 79 to 211 mm Hg during the experiments, which was considered high enough to arterialize. Animals should be maintained in a well‐perfused condition before “arterialization” and sampling of “arterialized” venous blood. In contrast, this is a limitation to the widespread use of this technique in diseased animals. For example, “arterialization” could not be used reliably for dogs in hypovolemic shock because of peripheral hypoperfusion. In these animals, AB gas should be analyzed to accurately assess oxygenation and acid‐base balance.

Capillary blood extracted from toenails^44^ and pinna margins^13^ can also be used to measure blood gases as an alternative to AB sampling. These sites are easier to access and have fewer complications.^12^, ^13^, ^44^, ^45^, ^58^ However, these sites are rarely used as air contamination might occur during blood collection.^16^ In comparison to capillary blood, peripheral venous blood sampling has the advantage of having no limit on the amount of blood collected, allowing for repeated analyses of many variables other than blood gas analysis, and avoiding air contamination.^16^ Lingual venous blood, which can also be used as a good substitute for AB for measuring blood gases,^42^ is not feasible in unanesthetized dogs.

In the present study, incandescent lamps were used to continuously heat paws, as in a previous study.^24^ However, this method appears impractical because the paws cannot be heated if the animal stands up or moves. Based on the present study results, further studies should be conducted to develop a practical, stable, and safe device to maintain the cutaneous temperature at 37°C in the dorsal part of the paw. In human studies, electric warming pads with a maximum temperature of 60°C were used.^1^, ^16^, ^20^ In these studies, the hand was heated by loosely bound pads that were separated from the skin by the sleeve of a bed jacket, and the cutaneous temperature was maintained at around 40°C for 10 to 15 minutes.^1^, ^16^, ^20^ We believe this technique is applicable to dogs.

Ideally, a venipuncture needle^59^ or cannula^60^ is introduced retrogradely, with the point directed toward the fingers; however, many earlier investigators did not mention this.^1^, ^16^, ^17^, ^20^, ^21^, ^24^ In the present study, venipuncture was not performed retrogradely, but sufficiently “arterialized” venous samples were obtained. More research is required to explore the significance of needle direction.

The findings of our study should be interpreted in light of several other limitations. First, the present study did not include data on venous blood samples collected from the nonheated paw. Therefore, the effect of heating on “arterialization” was not precisely evaluated. Additionally, general anesthesia might have strengthened the correlation and agreement between arterial and “arterialized” venous blood variables by reducing vasoconstriction via suppression of central thermoregulation,^20^ thereby inducing spontaneous “arterialization” of venous blood.^1^ Thus, in the present study, the strong correlation between the AB and “arterialized” venous samples might be because of the effects of multiple factors. However, the technique of continuously heating the paws appears to be the main factor, because a previous experiment in dogs under general anesthesia^24^ and a clinical study in human patients under anesthesia^20^ found that the pH, PCO_2_, and [HCO_3_^−^] values of heated venous blood were more comparable to those of AB than nonheated venous blood. Second, no compromised dogs were used in this study. Although a variety of acid‐base disorders were induced, the present study results might not accurately reflect the influence of metabolic and circulatory abnormalities on “arterialization” in diseased dogs. Third, the evaluation of the arteriovenous agreement using the criteria required to pass proficiency testing for quality control is based on human references because of the lack of similar criteria for dogs. The evaluation method has yet to be validated in veterinary medicine. However, to the best of our knowledge, there is currently no objective method for evaluating blood gas values in dogs using the Bland‐Altman plot. Fourth, the drugs used to maintain blood pressure and heart rate in our experiments might have influenced the study results. Ketamine increases heart rate, cardiac output, and AB pressure in healthy dogs^61^ at a constant infusion rate of ~10 μg/kg/min (plasma concentration of 2‐3 μg/mL in dogs^62^). Ketamine also causes mild but sustained vasodilation that is not dose‐dependent.^63^ In the present study, ketamine was administered at a rate of 10 to 60 μg/kg/min. As expected during our experiments, heart rate, systolic AB pressure, and mean arterial pressure were all close to or within the reference ranges in normal dogs (70‐120 beats/min, 120 mm Hg, and 87 mm Hg, respectively).^64^, ^65^ Succinylcholine chloride is thought to have transient and minor effects on the circulatory system. However, it was found to be a general vasodilator, with vasodilation caused in part by its local action on the peripheral circulation.^66^ This effect could have influenced our findings, particularly those obtained during induced respiratory acidosis with succinylcholine chloride administration. However, no bias in the data point distribution in Bland‐Altman plots for PCO_2_ between respiratory alkalosis (PCO_2_ < 37 mm Hg) and respiratory acidosis (PCO_2_ > 37 mm Hg; Figures 4D and 5D) was detected. The two drugs might help with “arterialization.” Fifth, in clinical settings, PCO_2_ values of the ASV might be influenced by underlying disease, stasis during sampling, and the degree to which the animal was maintained in a steady state. These conditions have an impact on local metabolism and perfusion. Finally, because of the limited number of dogs included in this study, our findings should be interpreted and generalized with caution.

The present study provided an ideal environment for collecting “arterialized” venous blood. Nevertheless, the PCO_2_ of the ASV had a systematic bias of +3 mm Hg when compared with the arterial value. The difference was within allowable limits. However, if bias is considered critical, the acid‐base status should be determined using AB samples or data calibrated to account for a bias of +3 mm Hg.

In conclusion, under experimental conditions, the ASV samples were more identical to the AB samples than the ACV samples for pH, PCO_2_, [HCO_3_^−^], and BE values in well‐perfused dogs. This effect might be associated with general anesthesia and ketamine infusion. Saphenous veins are more suitable for “arterialization” than cephalic veins. In stable, well‐perfused dogs, “arterialized” venous samples should be collected without venous stasis, with constant heating of the hind paw to achieve a cutaneous temperature of 37°C. In limited situations, “arterialization” can be used in clinical settings if a practical, safe, stable, and user‐friendly device for the heated‐paw technique is developed.

This technique could be an alternative to multiple AB gas samplings to evaluate blood gases, except for PO_2_. Although it has some limitations, pulse oximetry can help to compensate for this shortcoming. This method might be useful in cases where ketamine infusion is used for postoperative analgesia.

## CONFLICT OF INTEREST DECLARATION

Authors declare no conflict of interest.

## OFF‐LABEL ANTIMICROBIAL DECLARATION

Authors declare no off‐label use of antimicrobials.

## INSTITUTIONAL ANIMAL CARE AND USE COMMITTEE (IACUC) OR OTHER APPROVAL DECLARATION

Approved by the Tokyo University of Agriculture and Technology Institutional Laboratory Animal Care and Use Committee.

## HUMAN ETHICS APPROVAL DECLARATION

Authors declare human ethics approval was not needed for this study.
